# Identification of Lineage-Specific *Cis*-Regulatory Modules Associated with Variation in Transcription Factor Binding and Chromatin Activity Using Ornstein–Uhlenbeck Models

**DOI:** 10.1093/molbev/msv107

**Published:** 2015-05-04

**Authors:** Marina Naval-Sánchez, Delphine Potier, Gert Hulselmans, Valerie Christiaens, Stein Aerts

**Affiliations:** ^1^Laboratory of Computational Biology, Department of Human Genetics, University of Leuven, Leuven, Belgium

**Keywords:** Ornstein–Uhlenbeck, *cis*-regulatory evolution, FAIRE-Seq, *Drosophila* species, eye development

## Abstract

Scoring the impact of noncoding variation on the function of *cis*-regulatory regions, on their chromatin state, and on the qualitative and quantitative expression levels of target genes is a fundamental problem in evolutionary genomics. A particular challenge is how to model the divergence of quantitative traits and to identify relationships between the changes across the different levels of the genome, the chromatin activity landscape, and the transcriptome. Here, we examine the use of the Ornstein–Uhlenbeck (OU) model to infer selection at the level of predicted *cis*-regulatory modules (CRMs), and link these with changes in transcription factor binding and chromatin activity. Using publicly available cross-species ChIP-Seq and STARR-Seq data we show how OU can be applied genome-wide to identify candidate transcription factors for which binding site and CRM turnover is correlated with changes in regulatory activity. Next, we profile open chromatin in the developing eye across three *Drosophila* species. We identify the recognition motifs of the chromatin remodelers, Trithorax-like and Grainyhead as mostly correlating with species-specific changes in open chromatin. In conclusion, we show in this study that CRM scores can be used as quantitative traits and that motif discovery approaches can be extended towards more complex models of divergence.

## Introduction

Mutations in regulatory sequences can lead to the birth or disruption of transcription factor binding sites (TFBS) that can affect developmental gene networks and can lead to changes in spatiotemporal gene expression patterns (Simpson and Ayyar 2008; Kalay and Wittkopp 2010; Ordway et al. 2014). *Cis*-regulatory changes are considered important drivers of molecular and organismal phenotype divergence and have been associated with disease (Visel et al. 2009; Maurano et al. 2012). Therefore, deciphering regulatory changes leading to changes in chromatin activity and gene transcription is an important question in biology.

Comparative studies of gene expression across and within species have been used to study the impact of natural selection on gene regulation. Studies on yeast (Busby et al. 2011), *Drosophila* (Rifkin et al. 2003), and mammals (Caceres et al. 2003; Gilad et al. 2006; Brawand et al. 2011) have identified expression divergence, even between closely related species. However, these studies often lack a connection with the underlying regulatory sequences that may have caused the observed lineage-specific gene expression changes. An interesting approach aiming to connect *cis*-regulatory changes to expression changes is to include hybrids into the comparison. Such studies have been successfully applied to yeast (Tirosh et al. 2009; Emerson et al. 2010) and *Drosophila* species (Wittkopp et al. 2004; McManus et al. 2010; Coolon et al. 2014) and have provided insight into *cis*- and *trans*-regulatory changes (McManus et al. 2010; Suvorov et al. 2013; Coolon et al. 2014).

Another approach to study the divergence of gene regulation is to directly examine the activity at the level of chromatin, including transcription factor (TF) binding, histone modifications, or nucleosome positioning. Several genome-wide comparative studies of TF binding measured by ChIP-Seq across species have studied the tempo of TF binding turnover for several TFs and organisms (Borneman et al. 2007; Odom et al. 2007; Bradley et al. 2010; He et al. 2011; Schmidt et al. 2010, 2012). Recent efforts compared the layer of TF binding changes with changes in gene expression and found surprisingly few causative links, indicating a complex relationship between *cis*-regulatory changes, *cis*-compensatory changes, and gene expression changes (Paris et al. 2013; Wong et al. 2015).

Also, computational methods are often used to analyze the evolution of regulatory elements by comparing nucleotide sequence or motif composition across the phylogenetic tree. The conservation of noncoding regions across species (i.e., purifying selection) has been often used to identify regions with regulatory function and methods that use phylogenetic trees and evolutionary models have been used mainly to identify conserved elements. The phylogenetic tree can indeed be a useful instrument, even for the purpose of detecting conserved elements, because it allows assessing whether a given sequence is indeed under negative selection, or constraint, which is only possible when a multiple alignment and the phylogenetic distances are taken into account (Siepel et al. 2005). A widely used example where such an approach is used to identify conserved motifs across the genome is the Branch Length Score. By comparing the total branch length of the tree that underlies all the observed motif instances in the tips, highly informative sets of DNA words have been identified across yeast, *Drosophila*, and vertebrate genomes (Kellis et al. 2003, 2004; Xie et al. 2005; Kheradpour et al. 2007; Stark et al. 2007). Sequence conservation has also been a fruitful cue for motif discovery methods in sets of coexpressed genes (Ho Sui et al. 2007; Aerts et al. 2010; Gotea et al. 2010; Herrmann et al. 2012; Kwon et al. 2012; Janky et al. 2014).

Although functional *cis*-regulatory elements are under constraint, they show considerable turnover and are considered as a playground for evolution (Wray 2007; Carroll 2008). Certain nucleotide substitution models have been used to characterize turnover of TFBS. Moses et al. (2006) used a loglikelihood ratio between the Halpern–Bruno (HB) model and the Hasegawa–Kishino–Yano model to assess whether a TF binding site is conserved or not conserved, and found that approximately 5% of Zeste binding sites in *Drosophila* show turnover. Doniger and Fay (2007) also found that TF binding sites are frequently gained and lost, using a model of semiconservation, whereby a TF binding site may be conserved in some species (suggesting a putative function of the site), but not all. Another model, the McDonald–Kreitman test, considers polymorphisms within a species and substitutions between species (McDonald and Kreitman 1991) and has been used to assess TF binding site gains and losses in *Drosophila* (He, Holloway, et al. 2011), and was also applied to all human TFs with ENCODE ChIP-Seq data (Arbiza et al. 2013).

Although studying turnover of individual TF binding sites can provide interesting hypotheses, many studies have shown that enhancers and promoters can show substantial gains, losses, and reshuffling of TF binding sites, while maintaining a comparable regulatory activity (Ludwig et al. 2000; Dermitzakis and Clark 2002; Dermitzakis et al. 2003). A *cis*-regulatory module (CRM) can show divergence not only by changing the composition of TF binding sites but also by gains and losses of entire CRMs around a gene locus (Wittkopp and Kalay 2012). Several methods for CRM detection have taken the evolution of TF binding sites into account, by implementing evolutionary models of CRM turnover, such as cisEvolver (Pollard et al. 2006) and EMMA (He et al. 2009). Although these methods have been useful to identify CRMs in a reference species, to our knowledge, quantitative changes in CRM scores have not been used to identify divergent CRMs and consequent evolution of gene regulatory networks.

In this study, we investigate turnover of the entire CRM—the CRM score is used as quantitative trait—while allowing turnover of TF binding sites within a CRM. To model quantitative CRM changes we investigate the Ornstein–Uhlenbeck (OU) model, an extension of the Brownian motion (BM) model (Felsenstein 1988). Although the BM assumes that quantitative changes are caused by random drift, the OU model assumes that changes are the result of a shift toward a new optimum state (Bedford and Hartl 2009). This method was first used by Hansen (1997) to analyze the evolution of quantitative phenotypic traits, and recently has been applied to the evolution of gene expression (Brawand et al. 2011; Rohlfs et al. 2014). The OU model can be extended by defining different selective regimes (also called the Hansen model), whereby each regime states a priori which lineage is expected to have changes in the quantitative trait (Hansen 1997; Butler and King 2004; Hansen et al. 2008). After testing this model on CRM scores, we also ask whether this framework can be applied to identify divergent motifs/CRMs underlying set of coevolving genomic regions, analogously to (conserved) motif discovery on coexpressed genes or coregulated regions such as ChIP-Seq peaks. The joint analysis of the genome sequence, the chromatin landscape, and the emerging gene expression across species in a particular cell type and time point may provide means to further disentangle the evolution of functional enhancers and gene regulatory networks (Shibata and Crawford 2009; Connelly et al. 2014). We show that modeling CRM divergence can be an important component of such integrative comparative genomics studies.

## Results

### Applying OU Models to CRM Scores

**Fig. 1.**
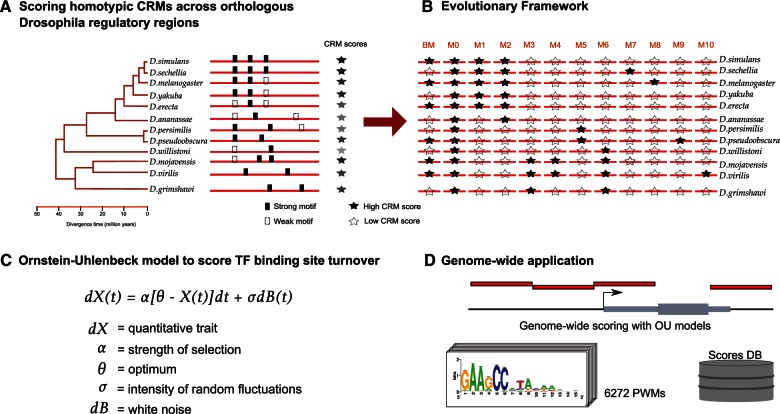
Application of the OU model at the CRM score level. (*A*) Calculation of CRM scores for a given PWM is performed with an HMM that aggregates over all possible matches to the PWM. A genomic region in *Drosophila melanogaster* is shown, alongside all the orthologous regions in the other 11 species (obtained from whole-genome alignments by liftOver). (*B*) The evolutionary scenarios considered are BM; M_0_: one global optimum across species; and nine models presenting a branch-specific optimum. M1: melanogaster subgroup optimum; M2: melanogaster group optimum; M3: *D. mojavensis*, *D. virilis,* and *D. grimshawi* shared optimum; M4: *D. mojavensis* and *D. virilis *shared optimum; M5: obscura group optimum; M6: *D. willistoni*, *D. mojavensis*, *D. virilis,* and *D. grimshawi *shared optimum; M7: *D. sechellia* specific optimum; M8: *D. melanogas*ter specific optimum; M9: *D. pseudoobscura *specific optimum; M10: *D. virilis *specific optimum. (*C*) The OU model formula assess the evolution of the quantitative trait X (here the CRM score) across phylogenetic time; d*X*(*t*) = α [θ − *X*(*t*)]d*t* + σ d*B*(*t*), where the term alpha (α) represents the action of selection; θ the optimum trait value, d*B*(*t*) is the ensemble of independent normally distributed random variables, and σ measures the intensity of the random fluctuations in the evolutionary process. (*D*) Application of the OU model across the entire *Drosophila* regulatory genome (136K regions, Herrmann et al*.* 2012) for all selective regimes and for a collection of 6,272 PWMs.

Our first aim was to develop a CRM prediction method to identify significantly divergent CRMs across a phylogeny of 12 sequenced *Drosophila* species. For a given input position weight matrix (PWM), we score a given genomic region in the reference species *D. melanogaster*, and all its orthologous regions in the 11 other genomes, with a Hidden Markov Model (HMM; see Materials and Methods). The HMM score represents a homotypic cluster of binding sites (although single PWM matches are also considered), and this score is used as quantitative trait (fig. 1*A*). Its distribution across the phylogenetic tree is then statistically assessed for whether it shows conservation, random drift, or selection in a particular lineage. To this end we use an extension of the OU model (Hansen 1997; Butler and King 2004; Hansen et al. 2008), considering 11 selective regimes (fig. 1*B* and *C*). The most simple regime is random drift, as modeled by BM (Felsenstein 1988). The second regime represents one global CRM score optimum across the phylogeny, or stabilizing selection, whereas the remaining regimes represent different branch-specific shifts in CRM score optima.

We first illustrate our approach on a previously known divergent enhancer, namely a CRM that controls *Dscam* expression and that is bound by Atonal (Aerts et al. 2010). The homologous enhancer in *D. virilis* has lost the Atonal binding site, and consequently its reporter activity (Aerts et al. 2010) (supplementary fig. S1, Supplementary Material online). To assess whether this enhancer adheres to a particular selective regime we performed the likelihood ratio (LR) test between each lineage-specific model (M_1__→__10_) against a model that represents one global optimum (M_0_), and compared with BM. The *Dscam* enhancer is predicted to diverge significantly, with a *D. virilis* specific loss, based on the LR between *D. virilis* specific loss (M_10_) and conservation (M_0_) (LR = 28.99; *P* = 7.25 × 10^−^^8^).

We also calculated the (weighted) Akaike Information Criterion (wAIC) for this example (Burnham and Anderson 2004; Schraiber et al. 2013). The AIC provides a correction on the LR when comparing models with a different number of parameters (e.g., BM is the simplest); and furthermore allows comparing models that are not nested. The *Dscam* enhancer has a higher wAIC score for M_10 _(0.99) versus M_0_ (3.73 × 10^−^^6^), and versus BM (4.31 × 10^−^^5^). Hence, the wAIC also suggests a *D. virilis* specific loss of the Ato binding sites at the *Dscam* enhancer (the model with the lowest AIC is chosen).

Finally, we tested the significance of this finding by a parametric bootstrapping approach (Boettiger et al. 2012) (see Materials and Methods), which confirmed that this region fits better to the *D. virilis* specific model compared with the model with one global optimum, and compared with BM (supplementary fig. S1, Supplementary Material online). Therefore, we conclude that the OU model can accurately detect, and quantify, this branch-specific loss of TF binding at a given CRM.

### Genome-Wide Prediction of Divergent CRMs Using AIC

**Fig. 2.**
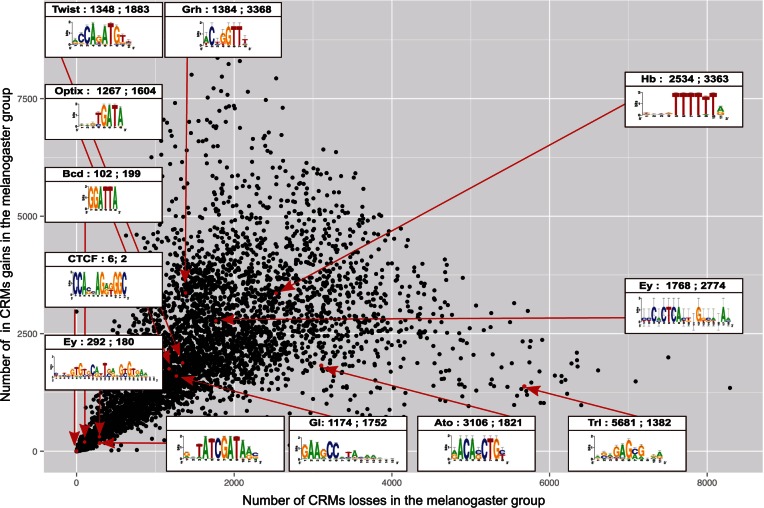
CRM turnover gains and losses per PWM in the melanogaster subgroup. *X* axis: Number of regions with a significant lower CRM score in the melanogaster group species for a collection of 6,272 PWMs. *Y* axis: Number of regions with a significant higher CRM score in the melanogaster group species for a collection of 6,272 PWMs. All divergent regions have a lower AIC for M2 versus M_0_. Several example PWMs are highlighted, including TFs involved in early embryogenesis (e.g., Hunchback, Bicoid, Twist), in eye development (e.g., Eyeless, Glass, Atonal), and several general factors (e.g., Trl, Dref, CTCF). Among the motifs with the lowest divergence are CTCF, p53, and Dref. Among the motifs with the highest number of CRM losses is Trl.

Next, we applied the OU model to 136K candidate regulatory regions covering the entire noncoding genome of *D. melanogaster* (Herrmann et al. 2012), for 6,272 position weight matrices (fig. 1*D*). Using the wAIC we compared each model (M_1__→__10_) versus conservation (M_0_). This way, we were able to define for each PWM, for each of the nine lineage-specific models, sets of candidate divergent regions as those with a higher wAIC compared with M_0_. Note that because these are pairwise comparisons, similar results can be obtained using the LR test, but the wAIC is more robust since a branch-specific shift model contains more parameters than the single-optimum model M_0_. In addition, the wAIC allows comparing multiple models at once, including BM (see Discussion). When we examined the resulting sets of AIC-based divergent CRMs, we observed that the size of each set is partly dependent on the information content of the PWM (fig. 2), corroborating earlier findings (Lusk and Eisen 2010). Thus, longer and less degenerate PWMs (i.e., with high information content) show lower turnover and less lineage-specific divergent regions independently of the evolutionary model. For example, the CTCF motif results in the lowest number (only 5) of divergent CRMs. Interestingly, we found no overall genome-wide bias toward either gains or losses of homotypic CRMs (fig. 2). Nevertheless, several individual TFs show a strong difference in the number of gained versus lost enhancers (fig. 2). In conclusion, CRM divergence scoring can be used to predict evolutionary gains and losses for thousands of position weight matrices.

### The *scratch* Gene Harbors Conserved and Divergent Glass Target Enhancers

**Fig. 3.**
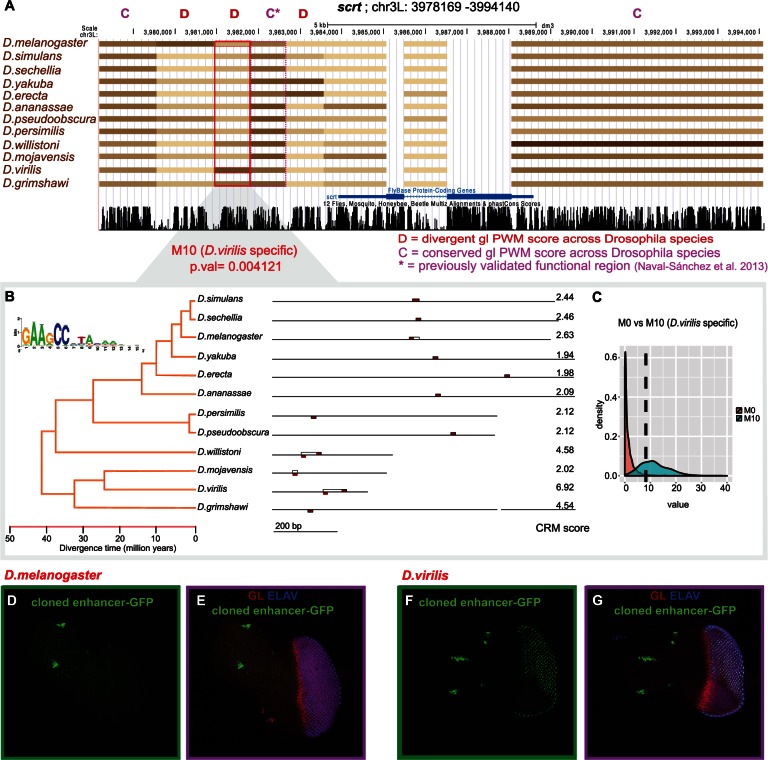
Application of OU predicts a de novo *scrt *enhancer in *Drosophila virilis*. (*A*) Predicted CRMs for the Glass PWM are color-coded: The darker the color the higher the CRM score. The highlighted region is predicted to be divergent, with a CRM gain in *D. virilis.* (*B*) Representation of the *Drosophila* phylogenetic tree. Orthologous *Drosophila* regions for regulatory region D, with a very high CRM score in *D. virilis* (6.92). The Glass motifs (red rectangles) in the detected highest scoring homotypic Glass CRM (open boxes) are shown. On the right, the CRM score. (*C*) Distribution of the LR statistic comparing M_0_ (conservation) versus M10 (*D. virilis* specific change), derived by parametric bootstrapping. (*D–G*) In vivo enhancer-reporter results for the *D. melanogaster* region and the *D. virilis* region, both tested in transgenic *D. melanogaster*. Antibody staining for GLASS (red), ELAV (blue), and GFP (green).

To investigate whether predicted divergent CRMs are also functionally divergent, we focused on the targets of one particular TF, namely Glass, a master regulator of photoreceptor differentiation (Moses et al. 1989; Ellis et al. 1993). Particularly, we identified all divergent Glass CRMs for seven previously validated target genes of Glass (*scratch *[*scrt*], *chp*, *retn*, dpr10, *CG6329*, *Lim3*, and *dmrt99B*) (Naval-Sánchez et al. 2013)*.* Out of the 138 regions (all regions in the 5 kb upstream and introns), 11 enhancers (9%) corresponding to five target genes (three to *scrt*, three for *Lim3, *three for *retn, *one for *dpr10*, and one for CG6329) presented a significant binding gain (CRM score in one species ≥ 6) in a lineage-specific manner. Note that this finding suggests that some Glass target genes contain apart from conserved enhancers also lineage-specific “shadow” enhancers (fig. 3*A*). For example, one *scrt *enhancer presents a higher CRM score (6.9) in *D. virilis *compared with the other species (2.62 on average) (fig. 3*A* and *B*). This difference can be explained by a *D. virilis* specific evolutionary change as suggested by the LR (LR = 8.23; *P* = 0.0041) and by a higher wAIC in the *D. virilis* specific model (M_10_, 0.89) compared with the conservation model (M_0_, 0.11). We also tested the significance of this finding by a parametric bootstrapping approach (Boettiger et al. 2012) (see Materials and Methods), showing that this region fits better to the *D. virilis* specific model compared with the model with one global optimum (fig. 3*C*). Also when compared with other models such as BM or an alternative branch-specific model M6 (gain in Dwil/Dmoj/Dvir/Dgrim), M_10 _was found to fit best (supplementary fig. S2, Supplementary Material online).

Next, we tested this predicted de novo enhancer using an in vivo enhancer reporter. Particularly, we created transgenic *D. melanogaster* flies carrying the *D. virilis* sequence and flies carrying the *D. melanogaster *sequence. We found that the *D. virilis* enhancer having a high Glass CRM score is active in photoreceptors whereas the *D. melanogaster* sequence has no activity in the eye imaginal disc (fig. 3*D*–*G*). Therefore, we present for the first time that the application of the OU model at the CRM level can lead to the de novo discovery of an enhancer.

### Motif Discovery Approach Based on Divergence: Application to Cross-Species ChIP-Seq and STARR-Seq Data

**Fig. 4.**
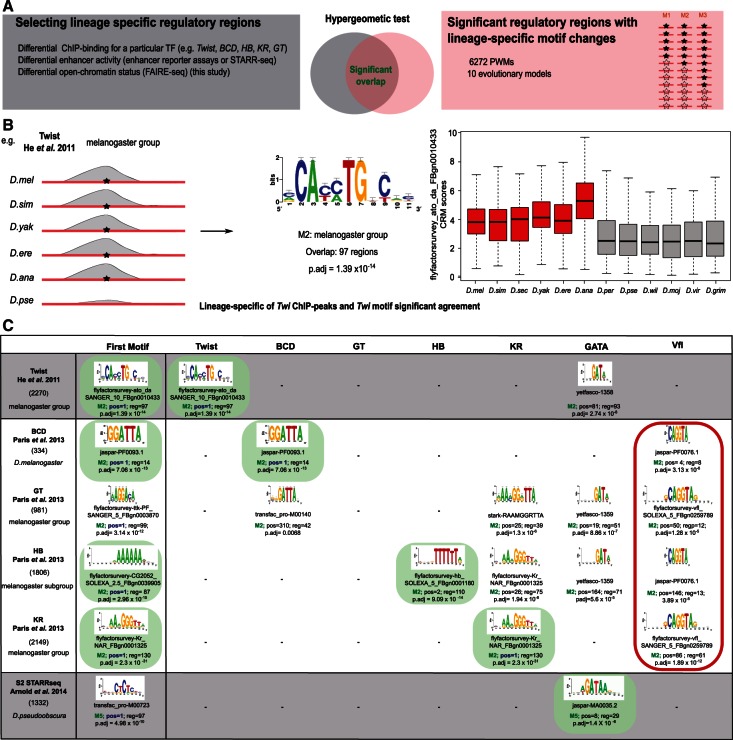
Application of divergent motif discovery to publicly available cross-species data sets. (*A*) Overview of the procedure. A set of divergent regions (left), obtained by cross-species ChIP-Seq, STARR-Seq, or FAIRE-Seq, is compared against all sets of predicted divergent regions (right) using a hypergeometric test. (*B*) Example analysis for divergent Twist ChIP-Seq peaks (He et al. 2011) (losses in *Drosophila pseudoobscura*; or gains in the melanogaster group), for which the most significant overlap was found for the Twist CRM gains in the melanogaster group. (*C*) All results for six data sets, including ChIP-Seq binding in the embryo for Twist (He et al. 2011), BCD, GT, HB, and KR (Paris et al. 2013) and functional enhancers specific for *D. pseudoobscura* detected by STARR-Seq in S2 cells (Arnold et al. 2014). Green motifs indicate the expected motifs to be found on the diagonal (all correct except Giant). Red border marks all the Zelda CRMs gains and losses, only found for the embryonic ChIP peaks.

Next, we asked whether our database with divergent regions for thousands of motifs could be used as a “divergent motif discovery” approach. The goal of such an approach would be to predict for which PWM the CRM divergence correlates with an observed functional divergence. This functional divergence could be, for example, a change in ChIP-Seq peaks, or a change in enhancer-reporter activity. To address this question we assessed the overlap between the input set of functional regulatory regions and each set of predicted divergent regulatory regions—gains and losses—for a particular PWM, using a hypergeometric test. This way we identify for a particular set of lineage-specific functional regions the most enriched lineage-specific gains or losses of a particular PWM and TF (fig. 4*A*).

To test this procedure, we first applied it to a public ChIP-Seq data set for Twist across six species, namely, *D. melanogaster*, *D. simulans*, *D. erecta*, *D. yakuba*, *D. anannassae,* and *D. pseudoobscura *(He, Bardet, et al. 2011). As in the original article, we selected regions with a Twist ChIP binding signal based on fold-change (greater than 2-fold) in lineages for which we have modeled a selective regime, such as the *D. melanogaster* subgroup, the *D. melanogaster* group, and the obscura group. Next, we tested the significance of the overlap between the Twist regions bound in a branch-specific manner and the regulatory regions considered under a branch-specific selective regime for any of the 6,272 PWMs (fig. 4*A* and *B*). We observe that regions with a gain of Twist binding in the melanogaster group (*D. melanogaster*, *D. yakuba,* and *D. ananassae *[2,270 regions]), the first predicted motif to have a selective shift in the CRM optimum is an E-box, representing the Twist motif (fig. 4*B* and *C*). Thus, a better lineage-specific CRM score of a Twist-associated PWM is related to regions with a quantitatively greater binding by ChIP-Seq. Interestingly, together with Twist*-*related PWMs other motifs appear that also represent a branch-specific shift in CRM scores for the species of interest. One such example is the Dorsal PWM (pos = 49, nb_reg = 206, *P*-adj = 4.65 × 10^−^^9^), a Twist cofactor (Zeitlinger et al. 2007), suggesting that Dorsal binding site turnover is correlated with turnover of Twist binding.

We also analyzed divergent binding peaks for Kr, Gt, Hb and Bcd across five *Drosophila* species, namely, *D. melanogaster, D. yakuba*, *D. erecta*, *D. pseudoobscura,* and *D. virilis* (Paris et al. 2013). For each of the 6,272 PWMs, we performed a hypergeometric test between species-specific ChIP-Seq peaks and the sets of regions predicted to follow the melanogaster group selective regime (fig. 4*A*). At the exception of Giant, for which its PWM shows no relation to lineage-specific binding, for all other TFs, the regions presenting higher binding have the corresponding TF motif with the most significant turnover (fig. 4*C*). As control we found that in most cases no unrelated TF was recovered, for example, Twist CRM changes do not affect the lineage-specific binding of the factors Bcd, Gt, Hb, and Kr. However, it is possible that the appearance of high-scoring Kruppel CRMs in a lineage-specific manner could influence a better binding of Giant and Hunchback in those lineages (fig. 4*C*). Finally, it is worth mentioning that for the lineage-specific binding of Bcd, Hb, Gt, and Kr we find many regions presenting a strong enrichment of the PWM of Zelda (Vfl) in the corresponding lineages (fig. 4*C*). The relation of Zelda binding, a zygotic chromatin modifier, affecting the binding of Bcd, Hb, Gt, and Kr has been previously reported (Bradley et al. 2010; Paris et al. 2013). Therefore, gain or loss of Zelda binding sites may agree with gain or loss of binding of these segmentation factors.

In a third case study, we analyzed species-specific enhancers detected by STARR-Seq in S2 cells (Arnold et al. 2014). This study identified lineage-specific enhancers, particularly 525 *D. melanogaster*, 515 *D. yakuba*, 632 *D. ananassae*, 1,332 *D. pseudoobscura*, 1,214 *D. willistoni*, 434 melanogaster subgroup, and 255 melanogaster group. In all these sets of divergent enhancers, we almost always find the Serpent (Srp) motif (GATAAG) in the correct species or branch of interest (fig. 4*C*). Srp expression has been shown to be relevant for S2 cell morphology and growth, and therefore the Srp cistrome is expected to be active in S2 cells (Rämet et al. 2002). For example, in the melanogaster group (235 regions) the GATA PWM was found as the most significantly lost motif in M3 (*D. mojavensis*, *D. virilis,* and *D. grimshawi*) (*P*-adj = 6.73 × 10^−^^5^) or for the *D. pseudoobscura* STARR-Seq specific regions, the GATA PWM is found in the *D. pseudoobscura* specific lineage (*P*-adj = 1.41 × 10^−^^6^) (fig. 4*C*). Altogether, these validation results indicate that the use of the OU model applied to a library of PWMs and across the entire genome can help to detect selective forces acting at the *cis*-regulatory level leading to alterations in enhancer functionality across lineages. These results also show that the correct TF can be identified, for which the binding sites show turnover in relation to enhancer function.

### Species-Specific Chromatin Changes in the *Drosophila *Eye Are Associated with Trithorax-Like and Grainyhead Binding Site Turnover

**Fig. 5.**
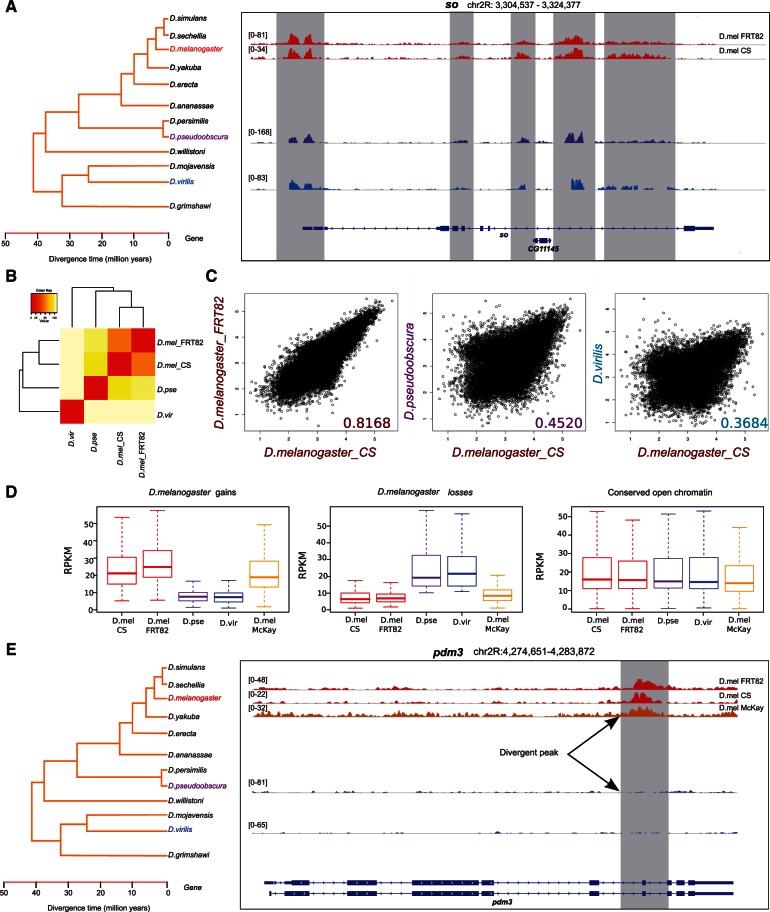
FAIRE-Seq open chromatin profiling in the eye disc of three *Drosophila* species. (*A*) Example gene (*sine oculis*) showing conserved FAIRE-Seq peaks. (*B*) Heatmap of spearman correlations of FAIRE-Seq signal across the *Drosophila* species *D. melanogaster CantonS*, *D. melanogaster FRT82*, *D. pseudoobscura,* and *D. virilis*. (*C*) Pairwise comparison of FAIRE-Seq signal between species, showing more divergence with more evolutionary time. Shown are the values for 19,424 peaks, filtered 1 RPKM in three species, length >150 bp. (*D*) Comparison of *D. melanogaster* gains and losses to an independent *D. melanogaster* on eye-antennal disc FAIRE-Seq sample (McKay and Lieb 2013). (*E*) Example of a *D. melanogaster* specific gain of an open chromatin peak in the *pdm3 *gene.

Next, we applied this framework to identify *cis*-regulatory variation underlying *Drosophila* eye development. To quantify enhancer activity genome-wide across different species, we made use of open chromatin profiling by FAIRE-Seq (Gaulton et al. 2010). We applied FAIRE-Seq to the eye-antennal imaginal disc of third instar wandering larvae of three species, namely *D. melanogaster*, *D. pseudoobscura,* and *D. virilis, *spanning 45 My of evolutionary divergence. Overall, we found that the open chromatin landscape is highly conserved between species, with 81.9% and 82.6% of the top 1,000 *D. melanogaster* peaks being recalled in the top 10,000 of *D. pseudoobscura* and *D. virilis,* respectively (the expected overlap conservation based on two different *D. melanogaster* strains is 98.7%; differences between strains might be due to technical errors or polymorphisms). As an example of this conservation, figure 5*A* shows the locus of the *sine oculis* gene, a key TF involved in retinal determination. Quantitatively, the peak heights correlate between the species, and are, as expected, more divergent with higher divergence time (*R*^2 ^= 0.452 with *D. pseudoobscura*, and *R*^2 ^= 0.368 with *D. virilis*, whereas *R*^2 ^= 0.817 between two *D. melanogaster *strains) (fig. 5*B* and *C*). The increasing chromatin activity divergence with phylogenetic distance may indicate that regulatory changes accumulate with species divergence time. Next, we categorized all the comparable peaks (i.e., *D. melanogaster* sequences that have orthologous sequences in the other species, and vice versa) into a group that is conserved across the phylogeny, and several groups with species-specific gains or species-specific losses (see Materials and Methods). We also confirmed that the observed differences with both strains of *D. melanogaster *can also be recapitulated by comparing with a third, previously published, FAIRE-Seq data set for *D. melanogaster *obtained in the same tissue (McKay and Lieb 2013) (fig. 5*D* and *E*).

**Fig. 6.**
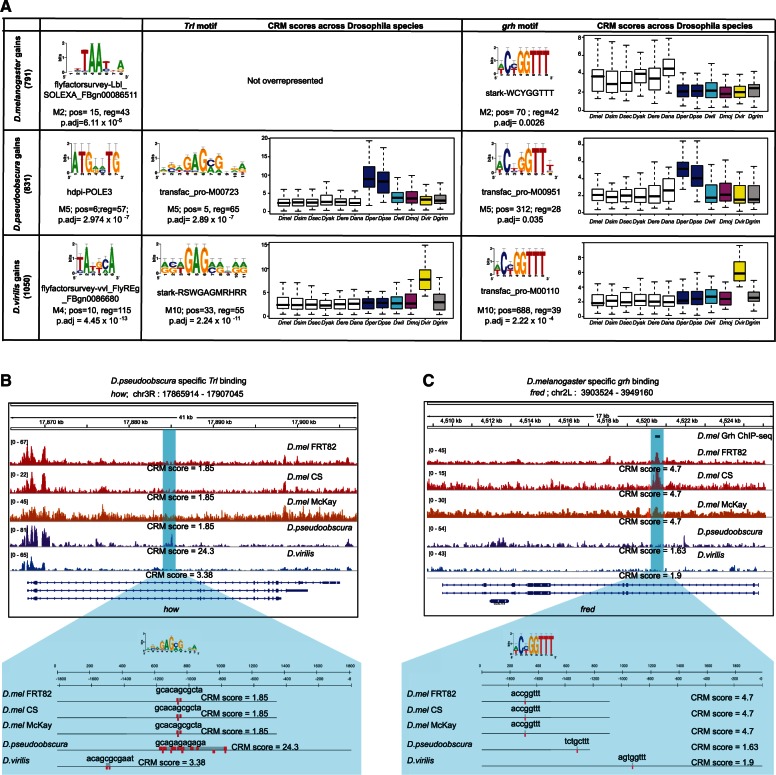
Lineage-specific Trl and Grh motifs are associated with lineage-specific open chromatin regions. (*A*) The rows represent FAIRE-Seq peak gains for each species. The first column shows the first and most significant PWM, for which CRM score changes overlap with open chromatin changes. The second column reports the position and *P* value of the Trl motif, and the third column the Grh motif. Trl and Grh CRM gains and losses correlate significantly with open chromatin changes. (*B*) Example of a Dpse-specific FAIRE-Seq peak, predicted to be gained because of a gain of a Trl CRM (increased CRM score from 1.85 to 24.3). (*C*) Example of a gene, *fred*, with a gain of a FAIRE-Seq peak in *Drosophila melanogaster*, predicted to be caused by a gain in Grh-binding sites. This position also contains a Grh ChIP-Seq peak (Potier et al. 2014).

Having identified sets of conserved versus species-specific open chromatin peaks, we first examined sequence constraint (PhastCons scores) in each group. We observed that open chromatin gains present lower sequence constraints compared with all detected peaks (Wilcox rank *P* = 0.028), whereas sequence conservation is only marginally different in the case of peak losses (Wilcox rank *P* = 0.056). Next, we analyzed the different groups of enhancers for divergent CRM scores, searching for TFs that may present CRM turnover in accordance with open chromatin changes. For that, we made use of the evolutionary framework presented in figure 1*B*. Most of the analyses identified enrichment of the motifs of two major TFs, namely Trithorax-like (Trl) and Grainyhead (Grh) (fig. 6*A*). Both these motifs appear with higher numbers and/or affinity in the species where the open chromatin peaks are higher (fig. 6*A*). For example, in the set of 115 peaks specifically gained in *D. melanogaster,* 42 regions present a greater Grh CRM score in the melanogaster group compared with the rest of *Drosophila* species (fig. 6*A*). The same is true for *D. pseudoobscura* and *D. virilis* gains which present a majority of regions with a significantly higher Grh CRM score in the obscura group and in the branch (*D. virilis*, *D. mojavensis*) or *D. virilis* only, respectively. Increased Trl binding scores on the other hand are overrepresented in *D. pseudoobscura* and *D. virilis* specific open chromatin peaks; and Trl CRM losses are enriched in *D. melanogaster* open chromatin losses (fig. 6*A*). These data indicate that gains in open-chromatin in a species-specific manner are associated with the de novo appearance of motifs for the chromatin regulators Trl and Grh in that species (fig. 5*A*). An example of such a case is the gene *how*, which shows a higher peak in *D. pseudoobscura* along with a higher Trl CRM score in that peak, only in the obscura species (CRM score is 24.3), whereas *D. melanogaster *and *D. virilis* present a much lower CRM score (1.85 and 3.38, respectively) (fig. 6*B*). Another example is the gene *fred*, for which an intronic region is accessible in *D. melanogaster* with a Grh binding score of 4.7, but shows reduced accessibility in *D. pseudoobscura* and *D. virilis* alongside a lower CRM score of 1.63 and 1.9, respectively. The hypergeometric analysis also identified other motifs besides those of the chromatin modifiers Grh and Trl. For example, for *D. melanogaster* gains a changing homeobox motif TAATTA was found, which could be potentially bound by *Lady bird late* or *Lim3.* Both these TFs are involved in the regulation of neurogenesis and could be therefore related to eye development. On the other hand, *D. virilis* peaks were enriched for corresponding CRM score changes for the motif of *ventral veins lacking*, a TF involved in axon guidance. In conclusion, evolutionary changes in open chromatin during eye development identify *cis*-regulatory sequence changes for broad chromatin regulators Trl and Grh.

### Validation of Grh Binding in *D. melanogaster* Specific Accessible Regulatory Regions

Grh was recently identified as a factor with strong overlap with open chromatin, suggesting a role as chromatin modifier (Potier et al. 2014). To examine whether predicted melanogaster specific Grh binding in *D. melanogaster* specific open-chromatin regions is functional, we used a Grh ChIP-Seq data set obtained in the same tissue (Potier et al. 2014). We assessed whether there is a significant overlap between *D. melanogaster* Grh ChIP-Seq regions (12928) and *D. melanogaster* specific open-chromatin (FAIRE-Seq) with predicted melanogaster Grh binding (OU model) (42 regions). This resulted in a significant overlap of 24 regions (*P* = 1.743 × 10^−^^14^). For example, the predicted CRM gain in the *fred* intron described above overlaps with a Grh ChIP peak (fig. 6*C*). As negative controls we performed the same test in *D. pseudoobscura* and *D. virilis* species-specific gains with predicted species-specific Grh gains. However, in both cases the overlap resulted in no significant overlap (*P* < 0.01). Thus, we conclude that predicted melanogaster specific Grh CRM gains within *D. melanogaster* specific open-chromatin are functionally relevant in *D. melanogaster* eye development.

## Discussion

*Cis*-regulatory changes are considered major drivers of lineage-specific characteristics. However, there is no straightforward method to identify *cis*-regulatory sequence changes underlying variation of gene regulation. We propose the use of OU models to infer selection at the *cis-*regulatory level underlying lineage-specific TF binding, chromatin activity, or gene expression. The use of OU processes to compare evolutionary models such as random drift, stabilizing selection or lineage-specific selection for quantitative morphological traits was first applied to the evolution of morphological traits (Hansen 1997; Butler and King 2004; Hansen et al. 2008). Later, these same models were applied to study the evolution of gene expression. These studies showed that stabilizing selection overall explains the observed variation in gene expression for the vast majority of genes involved in a biological process, much better than random drift can explain this variation (Bedford and Hartl 2009). Brawand et al. (2011) also employed OU models in a comparative analysis to assess the evolution of gene expression across six organs and ten mammals and found that gene expression dynamics varies among organs, lineages, and chromosomes. In our study, we extend the use of OU models to study the evolution of regulatory regions (e.g., promoters, enhancers), for which we consider homotypic CRM scores as the quantitative measure. These scores represent the total binding energy for a TF on a CRM, calculated by the factors PWM using an HMM (Frith et al. 2003). The HMM allows incorporating strong and weak binding sites, and accounts for the local clustering of binding sites within a confined genomic region. Therefore, our approach goes beyond the detection of individual TF binding site turnover. The variation of this CRM score is used by the OU model to estimate whether the CRM evolves under stabilizing selection with a single optimum across species or adaptive evolution with a distinct optimum in a particular branch of the phylogenetic tree.

We propose two strategies to implement the OU-based evolutionary framework at the CRM level. First, when there is prior knowledge of the motif or TF of interest, we use the OU model to identify divergent regions in the genome, or to assess the evolutionary mode of a particular set of sequences. We applied this principle to Atonal, and found a *D. virilis* specific loss of a *Dscam *enhancer; and to Glass, for which we found a de novo *scrt* enhancer in *D. virilis*. This shows that the application of the OU model at the CRM level can lead to the discovery of lineage-specific de novo enhancers, and as such it could also be applied to identify nomadic enhancers (Kalay and Wittkopp 2010). Second, when there is no prior knowledge of involved TFs, or of potential lineage-specific TFs, we use the OU model to identify candidate TFs by testing the CRM divergence rates for a large compendium of motifs. This technique can be considered a “divergent motif discovery” approach, in contrast to other existing “conserved motif discovery” approaches (Roider et al. 2007, 2009; Warner et al. 2008; Aerts et al. 2010; Gotea et al. 2010; Herrmann et al. 2012; Kwon et al. 2012; Janky et al. 2014). We have validated this strategy with publicly available data on *Drosophila* lineage-specific ChIP binding for several TFs, namely Twist (He, Bardet, et al. 2011), Bcd, Gt, Hb and Kr (Paris et al. 2013), and for functional enhancers identified by STARR-Seq (Arnold et al. 2014). In most cases, the correct TF can be identified, and shows overrepresented lineage-specific changes of its CRM score, in the set of divergently bound regions. In addition, often we detected the influence of lineage-specific binding of cofactors that can affect the binding of the TF of interest. For example, we found that Zelda binding can be lineage-specific and may underlie variation in binding of Bcd, Gt, Hb, and Kr. This finding is in agreement with previous studies (Bradley et al. 2010; Paris et al. 2013). It is also worth mentioning that for some factors, such as Twist, species-specific ChIP’d regions can present slightly different associated PWMs for the TF under study, thus suggesting that changes in the TF domain can affect evolution (Cheatle Jarvela et al. 2014).

In our genome-wide enrichment analyses, we considered sets of lineage-specific regions that are selected based on a higher wAIC score for the corresponding lineage-specific model versus a model that represents one global optimum. We obtained similar results using a threshold on the LR *P* value (unadjusted *P* value < 0.05) (data not shown). However, the advantage of wAIC is that it corrects for the difference in the number of parameters between models and it allows comparing multiple models at once, including models that are not nested. Thus, alternative model comparisons such as (M_1__→__10_), M_0_, and BM, or all the evolutionary models presented in figure 1*B* can be performed. When such alternatives are used to select divergent CRMs, we find that the divergence predictions become more stringent, resulting in smaller sets of predicted divergent CRMs under a particular evolutionary regime. Therefore, even if the enrichment of previous TFs is maintained, the overlap with sets of experimentally identified regions showing evolutionary divergence is less significant (supplementary figs. S3 and S4, Supplementary Material online). Thus, although the specificity may increase when comparing all models at once, we find that the enrichment analyses are less performant (likely due to the drop in sensitivity), compared with using pairwise comparisons between lineage-specific shifts versus conservation.

Finally, identifiability issues and lack of statistical power to discern between alternate models is an important concern in evolutionary models (Boettiger et al. 2012; Ho and Ané 2014). For example, when the simple BM is included in the comparison, many regions are assigned to BM rather than to a branch-specific shift model. However, further inspection by parametric bootstrapping shows that often this is due to the lack of power to assign some regions to particular models (supplementary fig. S5, Supplementary Material online). A related issue that others have reported concerns the use of AIC or other extended statistics for model choice such as the Bayesian Information Criterion, because they may favor models with a higher number of parameters (Ho and Ané 2014). Here, we did not encounter obvious biases toward any lineage-specific model (supplementary fig. S6, Supplementary Material online).

We analyzed CRM divergence underlying species-specific active chromatin activity across three *Drosophila* species and asked whether there exist overrepresented lineage-specific CRM changes leading to changes in open chromatin, or whether alternatively, variation in open chromatin is rather governed by “spurious” *cis*-regulatory changes without any clear enrichment for a particular motif. These analyses indicate that the de novo appearance of binding sites for chromatin-related factors such as Trl and Grh is correlated with species-specific chromatin opening, suggesting that the appearance in a particular lineage of a chromatin remodeler sequence such as Trl or Grh can influence the binding of these factors in that particular lineage and facilitate the accessibility or appearance of a new regulatory region in a given biological process. Interestingly, we do not observe any correlation between lineage-specific TFBSs for eye-master regulators such as Eyeless and Sine Oculis, with lineage-specific active chromatin. This may indicate that the turnover rate of the binding sites for the master regulators of eye development is relatively low (fig. 2), and that the core of the eye developmental gene regulatory network is conserved up to the *cis*-regulatory sequence level. On the other hand, our chromatin profiling data are obtained for the entire tissue, and do not allow assessing evolutionary changes of CRMs that are active in a small subset of cells.

Here we have focused our analyses on genomic regions, but a similar approach could in principle also be applied to sets of divergently expressed genes (Brawand et al. 2011), which could be an interesting future avenue. Motif discovery in gene sets is more difficult than in enhancer sets because of its larger and noisier search-space (e.g., 5 kb upstream and introns) relative to ChIP’d regions.

Altogether we present for the first time the use of OU processes to model CRM evolution and score correlations between changes in *cis* with observed divergent functional regions across species derived from ChIP-Seq experiments, open chromatin, histone modifications, enhancer-reporter assays or gene expression. The use of this method can be useful to answer questions about *cis*-regulatory evolution such as how often an enhancer is created or which TFs present higher turnover rates. In addition, even if changes in *trans* are thought to be selected against, a change in the expression optimum for a particular lineage can trigger changes in expression to its direct and indirect targetome. Although *trans* effects underlying divergent gene expression can be predicted by the classical approaches of conserved motif discovery, we here propose the complementary analysis of identifying motifs with *cis *effects, thus leading to a better understanding of the mechanisms underlying regulatory evolution.

## Materials and Methods

### Fly Stocks

The following fly stocks were used in this study: *D. melanogaster* Canton-S and FRT82 for FAIRE-Seq analysis. For nonreference species, namely, *D. pseudoobscura *and *D. virilis* we used the sequenced strains, obtained from the San Diego Stock Center (stock number 14011-0121.94 and 15010-105.118, respectively). All flies were raised at 25 °C on standard fly food.

### FAIRE-Seq Library Preparation

FAIRE-Seq was performed in accordance to Giresi et al. (2007) with some modifications. In brief, eye-antennal imaginal discs were dissected in PBT and cross-linked in 4% formaldehyde for 10 min. Chromatin was isolated using a three-step lysis protocol and the chromatin was subjected to a ten-cycle sonication (30 s on/off) to obtain fragments of 200–500 bp. Next, nucleosome depleted chromatin regions were extracted with phenol/chloroform followed by ethanol precipitation and RNAse A treatment. Finally, libraries were prepared according to the TruSeq protocol with indexes, pooled, and sequenced on Illumina HiSeq2000.

### Sequence Reads Quality Check and Mapping

Reads containing residuals of adapters sequences were discarded (FastX clipper version 0.013 with option -M15). Quality control assessment on the reads was performed using the software FastQC (version 0.9), mainly we checked for reads quality score (PHRED quality > 20) and primers contaminations. Reads passing the filtering were mapped against their respective genomes, namely *D. melanogaster* FlyBase genome release 5, *D. pseudoobscura* release 2, and the rest of nonreference species release 1. FAIRE-Seq reads were mapped using Bowtie2 (Langmead and Salzberg 2012).

### FAIRE-Seq Analysis across Species

Nonreference species alignments were converted to *D. melanogaster* coordinates using the liftOver tool from UCSC (parameters minMatch = 0.1) (Fujita et al. 2010). For each sample, peaks were called in *D. melanogaster* genome coordinates with F-Seq (Boyle et al. 2008) parameters-l = 200. F-Seq calculates the continuous read density estimation and regulatory regions (peaks) as the locations presenting greater than user defined standard deviation over the mean local background. We gathered the peaks called in each sample approximately 30,000 peaks for *D. melanogaster* strains, CS and FRT82, *D. pseudoobscura,* and *D. virilis*. Regions with 0 counts in one species were excluded in order to avoid differences in peak intensity caused by artifacts such as low mappability regions in a particular species. As a measure of chromatin accessibility we calculated the number of reads falling in a particular peak by HTseq counts (strn = no). Peak intensity was normalized following a within and between normalization procedure as with gene expression.

To identify open chromatin changes in particular lineages, we compared a model that assumes a single optimum expression level (OU model) for a given genomic region in all branches of the phylogeny with a model in which the region presents a novel optimum in a specific lineage (extension of the OU model “Hansen model”). We utilized the R package ouch (King and Butler 2009) to generate the following evolutionary regimes scenarios: A global optimum among the three species with open chromatin data, and each selective regime where one species has a distinct evolutionary optimum for chromatin accessibility. We correct by multiple testing using Benjamini and Hochberg corrections for the number of regions and number of models tested. To assess which selective regime (model) was explaining individual chromatin activity profiles the best, we used the LR between selective regimes against one shared global optimum across species regime. This resulted in a list of regions with significant activity changes across species.

### Sequence Divergence

The average PhastCons scores on the 12 flies, mosquito, honeybee, and beetle multiz alignment were used as a measure of conservation (Margulies et al. 2003) of a particular region.

### The OU Model

BM represents a neutral model of evolution where differences in traits are caused by drift. The equation representing BM is d*X*(*t*) = σ d*B*(*t*), where d*B*(*t*) is the ensemble of independent normally distributed random variables and σ measures the intensity of the random fluctuations in the evolutionary process. Alternatively, OU models build on the BM including a deterministic part of the equation triggered by the action of natural selection in a quantitative trait optimum across a phylogeny. OU is defined by the following equation: d*X*(*t*) = α [θ − *X*(*t*)]d*t* + σ d*B*(*t*). In here, the evolution of the quantitative trait X across phylogenetic time is not only dependent to random variances and their fluctuations. The term α [θ − *X*(*t*)] presents the terms alpha (α) considered as the action of selection on a trait and θ the optimum trait value. Thus, selection is influenced by the difference between the optimum trait value and *X*(*t*). If there is no selection α = 0, then we fall back to BM. The OU model reflects stabilizing selection. Alternatively, extensions of this model by hypothesizing distinct optima in different phylogenetic branches allow formulating different evolutionary hypotheses and test which model explains best the distribution of the data.

### Evolutionary Motif Analysis

*Drosophila melanogaster* genome has been previously partitioned into 136K nonoverlapping regulatory regions, based on Phastcons score, class I insulator binding, and excluding exon coding regions (Aerts et al. 2010; Herrmann et al. 2012). Orthologous regions in the rest of 11 *Drosophila* species were selected in accordance to liftOver (-minMatch = 0.1, multiple). All regions were scored for the presence of homotypic motif clusters using the program Cluster-Buster (Frith et al. 2003) for a collection of 6,272 PWMs. Cluster-Buster is a probabilistic approach, based on HMMs, which provides the loglikelihood that a region is a TFBS cluster compared with background. For each PWM, we hypothesized ten different selective regime scenarios in accordance to the *Drosophila* phylogenetic tree. These are BM, M_0_: the existence of a global optimum across 12 *Drosophila* species, and nine alternative models with different selective regimes along the *Drosophila* tree, namely M1: melanogaster subgroup species; M2: melanogaster group species; M3: *D. mojavensis*, *D. virilis,* and *D. grimshawi*; M4: *D. mojavensis* and *D. virilis*; M5: obscura group; M6: *D. willistoni*, *D. mojavensis*, *D. virilis,* and *D. grimshawi*; M7: *D. sechellia*; M8: *D. melanogas*ter; M9: *D. pseudoobuscura*; M10: *D. virilis*. To model this selective scenario framework, we made use of the R package ouch (King and Butler 2009). To assess which selective regime explains the best the evolution of a particular PWM in a particular regulatory region across species, we used the LR between a lineage-specific selective regime M_1__→__10_ against M_0_ (one shared global optimum across species), per model and region.LRs between M_0_ and M_1__→__10_ follow approximate a X^2^ (df = 1) from which a *P* value is derived. At the same time we computed the standard AIC (Burnham and Anderson 2004; Schraiber et al. 2013), which contrary to LR, corrects for the number parameters that need to be estimated as well as allowing multiple model comparisons and comparisons across nonnested models. Based on AIC we calculated the differences between models with the lowest AIC and the rest (Δ*_i_* = AIC*_i_* − min AIC) and normalized across models wAIC*_i_* = exp(−0.5*Δ*_i_*)/Σ^R^*_r_*_=1_ exp(−0.5*Δ*_i_*). That is the exponential of AIC variation of that model (Δ*_i_*) divided by the sum of the relative likelihoods for all candidate models, known as Akaike weights. The use of wAIC is a straightforward method for model selection and allows us to compare branch-specific models (M_1__→__10_) versus M_0_ and BM as well as a global comparison of all the models depicted in figure 1*B* at once. This way, we produced a list of *Drosophila* regulatory regions per TF that are significantly better explained (greater Akaike weight) by an alternative evolutionary model rather than a conserved evolutionary optima, conservation or drift and or, finally compared with the evolutionary scenario proposed in figure 1*B*.

We tested four different databases with sets of divergent regions: 1) Pairwise comparison of M_x _against M_0_ using the LR; 2) pairwise comparison of M_x _against M_0_ using the wAIC; 3) comparison of BM, M_x_, M_0_; and 4) comparison of all models. We have mainly used the pairwise comparison using the wAIC, but also provide the validation results for (3) and (4) in supplementary figures S3 and S4, Supplementary Material online.

Importantly, this is not the only measure used to identify a significant change, because we need in addition to have a significant overlap test (hypergeometric) between 1) input regions considered as functional in a lineage-specific manner, and 2) regulatory regions considered under a particular lineage-specific model. The hypergeometric distribution parameters used are the following:

Population size (*N*) = the number of regulatory regions based on Herrmann et al. (2012) genome cut (136,353 regions). Number of items in the sample (*n*) = number of regions considered as lineage-specific by experimental analysis such as ChIP, STARR-Seq, or FAIRE-Seq. Number of items in the population that are classified as successes (*K*) = number of regions in the population that are classified as lineage-specific by an evolutionary model (M_1__→__10_). Number of items in the sample that are classified as successes (*k*) = number of regions considered by experimental analysis as functional lineage-specific and also considered as lineage-specific under a tested lineage-specific evolutionary model (M_1__→__10_). Hypergeometric test *P* values are corrected for multiple testing by Bonferroni correction.

### Phylogenetic Monte Carlo

To assess the power of model selection for particular regions and a specific TF, we have used a parametric bootstrapping approach using the R package “Phylogenetic Monte Carlo” (pmc) (Boettiger et al. 2012). In brief, model parameters are derived from the user quantitative data across the phylogeny for model *a*. Next, 1,000 data sets are simulated under that model with its estimated parameters. For each set, the loglikelihood ratio δ = 2(log *L*_a_ − log *L*_b_) was calculated comparing model *a* and *b*. Next, the original data are used to estimate parameters under model *b* and 1,000 parametric bootstrap sets are generated. We obtain a set of δ for that model. Plotting the distributions of δ across both models we can observe where in the distribution the original LR falls. Thus, how likely is that the original data come from the derived particular model (Boettiger et al. 2012). The amount of overlap between both distributions shows the power of model choice; the greater the overlap the lower the power.

### Lineage-Specific ChIP-Seq Data Sets

Data sets for lineage-specific ChIP-binding for *Twist* TF were extracted from supplementary table S11, Supplementary Material online (He, Bardet, et al. 2011). We considered as *Twist* lineage-specific binding regions those presenting a ChIP binding score greater than 2-fold compared with the rest of species. The selected sets were *D. melanogaster* specific regions (166), *D. simulans* (82), *D. erecta* (247), *D. yakuba* (160), *D. ananassae* (760), and *D. pseudoobscura* (745). Finally, we also considered melanogaster subgroup regions (*D. melanogaster* and *D. yakuba*) and melanogaster group (*D. melanogaster*, *D. yakuba,* and *D. ananassae*) as those presenting 2-fold-change enrichment versus *D. pseudoobuscura*, 1,129 and 2,270 regions, respectively.

Species ChIP-Seq data from TFs *BCD*, *GT*, *KR,* and *HB* (Paris et al. 2013) were downloaded from GEO GSE50773. We selected regions for the melanogaster group species with a fold-change greater than 1 for the ChIP normalized data presented in the accessible metatable.

### Enhancer-Reporter Assays STARR-Seq Data Set

Lineage-specific enhancers were detected as in Arnold et al. (2014). Processed data were kindly provided by the STARK lab. We defined as lineage-specific enhancers as those with *P* ≤ 0.001 and ≥ 3-fold enrichment over input only in that particular lineage.

### Scrt Enhancer-Reporter Assays

Enhancer region containing the predicted glass-binding motif was polymerase chain reaction-amplified from genomic DNA of *D. melanogaster* and of *D. virilis* and cloned into the phiC31 and Gateway compatible reporter vector pH-attB-Dest (Aerts et al. 2010), injected into VK37 (Venken et al. 2006) by Genetivision, and crossed together to generate homozygous stocks.

### Immunohistochemistry

Eye-antennal imaginal discs at the stage of third instar larva were dissected and processed as described (Wang et al. 2002). Antibodies used were anti-GFP (Invitrogen) and the antibody against ELAV raised by G.M. Rubin was obtained from the Developmental Studies Hybridoma Bank, developed under the auspices of the NICHD, and maintained by The University of Iowa, Department of Biology (Iowa City, IA). Finally, we used phalloidin (Alexa Fluor 488).

### Data Access

FAIRE-Seq data (three species) are available from GEO (accession number GSE59706). Tables for each motif and for all *Drosophila* regions (see Materials and Methods) with AIC and wAIC scores for all the 11 evolutionary models (fig. 1*B*) can be accessed at our lab website (http://www.aertslab.org, last accessed May 13, 2015) under the Resources section.

## Supplementary Material

Supplementary figures S1–S6 and tables S1–S11 are available at *Molecular Biology and Evolution* online (http://www.mbe.oxfordjournals.org/).

Supplementary Data
