# Supplementary material for: Identification of Lineage-Specific Cis-Regulatory Modules Associated with Variation in Transcription Factor Binding and Chromatin Activity Using Ornstein–Uhlenbeck Models
Source: Mol Biol Evol. 2015 May 4;32(9):2441–55. doi: 10.1093/molbev/msv107 (PMC4540964; doi:10.1093/molbev/msv107)
Supplement: Supplementary Data [file supp_32_9_2441__index.html]

Identification of Lineage-Specific Cis-Regulatory Modules Associated with Variation in Transcription Factor Binding and Chromatin Activity Using Ornstein–Uhlenbeck Models — Identification of Lineage-Specific Cis-Regulatory Modules Associated with Variation in Transcription Factor Binding and Chromatin Activity Using Ornstein–Uhlenbeck Models — Supplementary Data 

# Identification of Lineage-Specific *Cis*-Regulatory Modules Associated with Variation in Transcription Factor Binding and Chromatin Activity Using Ornstein–Uhlenbeck Models

## Supplementary Data

files

**Files in this Data Supplement:**

- Supplementary Data - pdf file
