# Supplementary material for: Identification of Lineage-Specific Cis-Regulatory Modules Associated with Variation in Transcription Factor Binding and Chromatin Activity Using Ornstein–Uhlenbeck Models
Source: Mol Biol Evol. 2015 May 4;32(9):2441–55. doi: 10.1093/molbev/msv107 (PMC4540964; doi:10.1093/molbev/msv107)
Supplement: Supplementary Data [file supp_msv107_NavalSanchez_Supplement_R2.pdf]

Supplementary Material to:

Identification of lineage-specific *cis*-regulatory modules associated with variation in transcription factor binding and chromatin activity using Ornstein-Uhlenbeck models

Marina Naval-Sánchez, Delphine Potier, Gert Hulselmans, Valerie Christiaens and Stein Aerts

Laboratory of Computational Biology, Department of Human Genetics, University of Leuven, 3000 Leuven, Belgium

Correspondence: stein.aerts@med.kuleuven.be

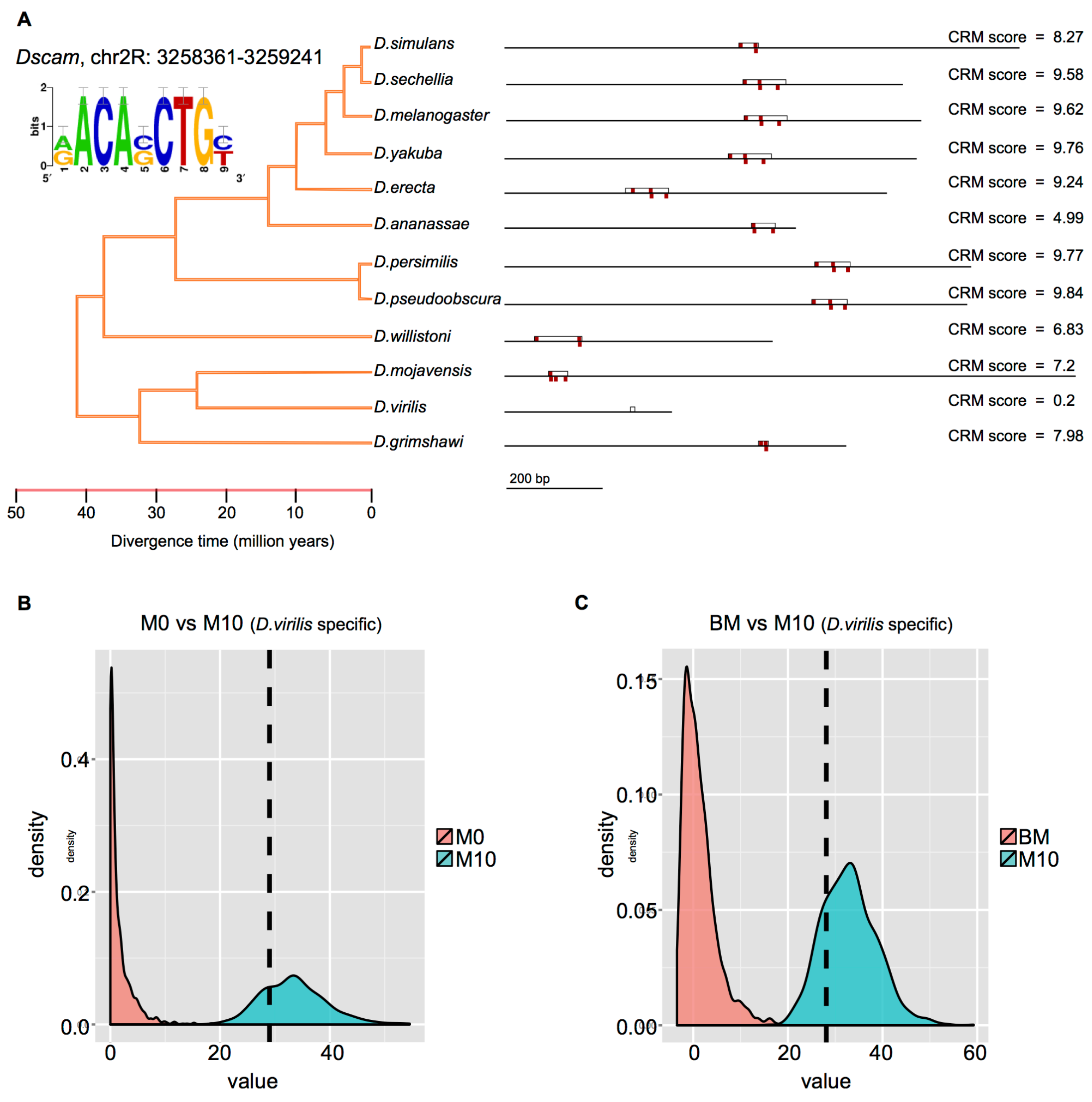

Supplementary Figure 1: Evolution of Atonal CRM scores in a *Dscam* regulatory region. (A) Representation of the Drosophila phylogenetic an orthologous regions of the enhancer (dm3 location: chr2R:3258361-3259241). The Atonal motifs (PWM stark-RACASCTGY) are represented in red for the highest scoring homotypic Atonal CRM. On the right the CRM score is shown. (B-C) Distribution of the likelihood ratio statistic for two different model comparisons. In blue, the distribution of the likelihood ratio values obtained by bootstrapping under the *D.virilis* specific evolutionary model, and in pink the distribution under M0 (conservation) (B) or random drift BM (C). 1000 replicates are simulated for each model. The dashed vertical line indicates the observed value of likelihood ratio when the models are fit for the *Dscam* divergent region. (B) M0 (conservation) *vs* M10 (*D.virilis* specific), (C) BM *vs* M10 (D.virilis): In both comparisons there is no overlap between distirbutions and the original log likelihood ratio falls clearly in the range of the M10 *D.virilis* specific model.

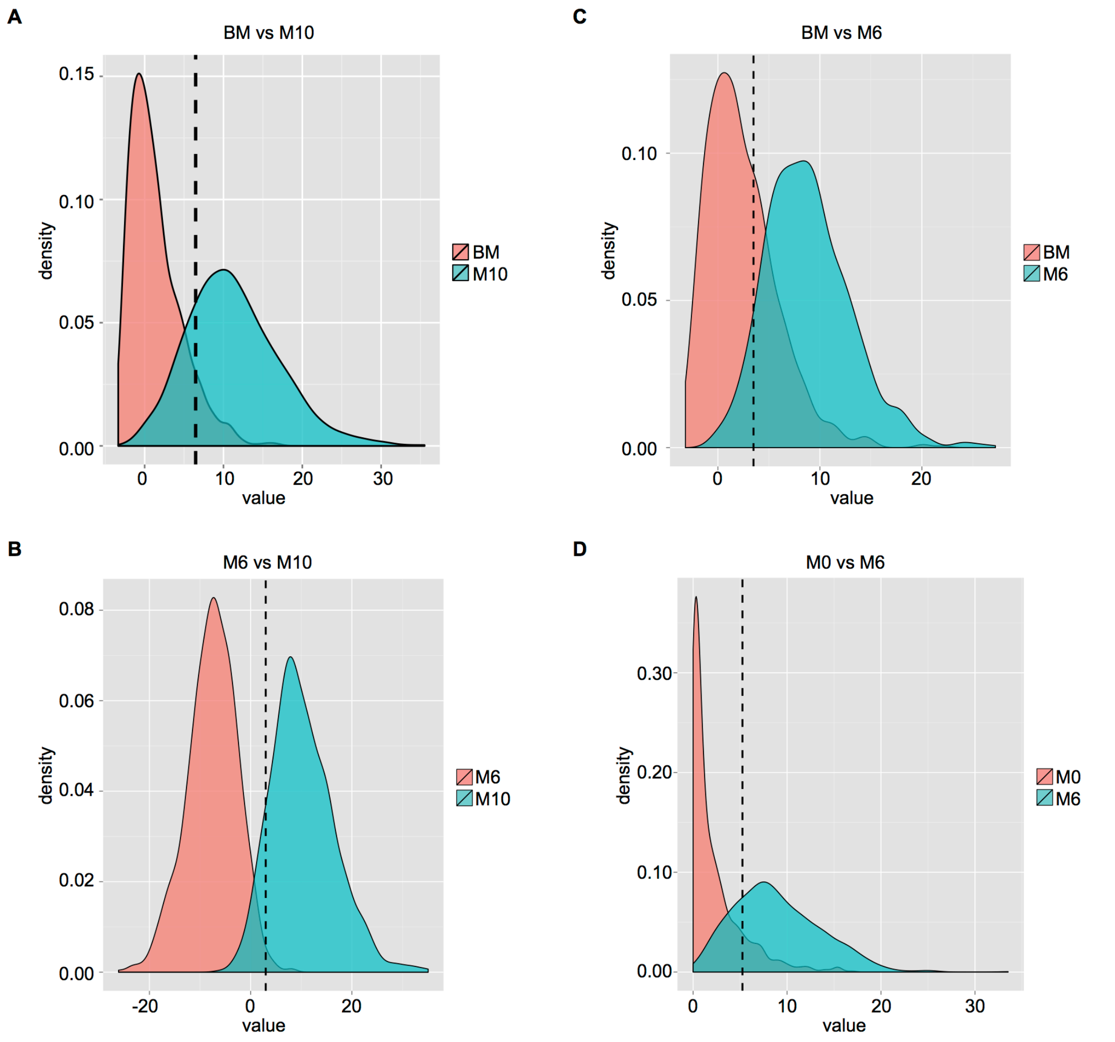

Supplementary Figure 2: Examination of model choice statistic for the evolution of Glass CRM scores in a regulatory region upstream of *scrt* (dm3 location: chr3L:3980929-3981800). Distribution of the likelihood ratio statistic for four different model comparisons. The dashed vertical line indicates the observed value of likelihood ratio when the models are fit for the *scrt* divergent region. (A-B) In each case the pink distribution shows the distribution of the likelihood ratio values obtained by bootstrapping under the simpler of the two models, namely Brownian Motion (BM), while the blue distribution shows the distribution under the more complicated of the two models. (A) BM *vs* M10 (*D.virilis* specific); (B) M6 vs M10 *D.willistoni, D.mojavensis, D.virilis, D.grimshawi* specific; (C) BM vs M6; (D) M0 vs M6. In A and B the observed ratio falls clearly in the range of the M10 *D.virilis* specific model. We can conclude that this results supports M10.

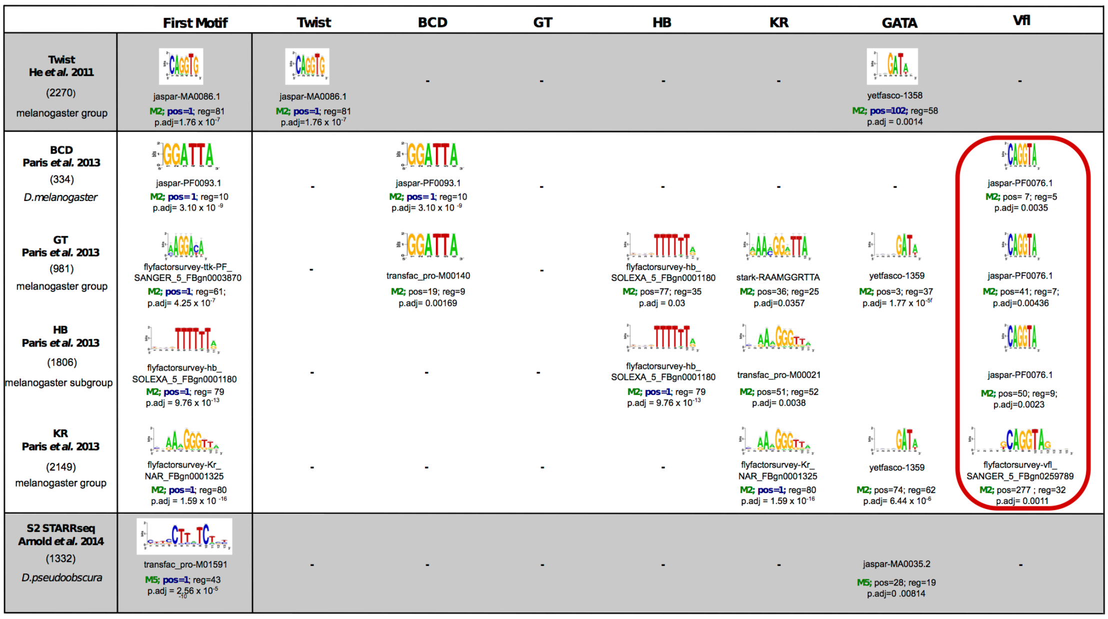

Supplementary Figure 3: Divergent motif discovery comparing three models with the wAIC: lineage-specific models versus conservation and BM, applied to publicly available cross-species data sets. All results for six datasets, including ChIP-seq binding in Drosophila embryo for Twist (He et al. 2011), BCD, GT, HB and KR (Paris et al. 2013) and functional enhancers specific for *D.pseudoobscura* detected by STARR-seq in S2 cells (Arnold et al. 2014). Green motifs indicate the expected motifs to be found on the diagonal (all correct except Giant). Red border marks all the Zelda CRMs gains and losses, only found for the embryonic ChIP peaks.

	First Motif	Twist	BCD	GT	HB	KR	GATA	Vfl
**Twist**<br>**He *et al.* 2011**<br>(2270)<br>melanogaster group	jaspar-MA0086.1<br>M2; pos=1; reg=51<br>p.adj=1.69 x 10^{-6}	jaspar-MA0086.1<br>M2; pos=1; reg=51<br>p.adj=1.69 x 10^{-6}	-	-	-	-	elemento-GATAAGC<br>M2; pos=26; reg=29<br>p.adj= 0.0258	-
**BCD**<br>**Paris *et al.* 2013**<br>(334)<br>*D.melanogaster*	jaspar-PF0093.1<br>M2; pos=1; reg=7<br>p.adj= 2.04 x 10^{-6}	-	jaspar-PF0093.1<br>M2; pos=1; reg=7<br>p.adj= 2.04 x 10^{-6}	-	-	-	-	-
**GT**<br>**Paris *et al.* 2013**<br>(981)<br>melanogaster group	ftransfac_pro-M00971<br>M2; pos=1; reg=34;<br>p.adj= 2.11 x 10^{-5}	-	jaspar-PF0093.1<br>M2; pos=3; reg=8<br>p.adj= 4.19 x 10^{-4}	-	flyfactorsurvey-hb_<br>SOLEXA_5_FBgn0001180<br>M2; pos=11; reg=23<br>p.adj = 0.012	-	yetfasco-1359<br>M2; pos=5; reg=23<br>p.adj= 8.02 x 10^{-4}	-
**HB**<br>**Paris *et al.* 2013**<br>(1806)<br>melanogaster subgroup	transfac_pro-M00234<br>M2; pos=1; reg= 29<br>p.adj = 5.42 x 10^{-6}	-	-	-	flyfactorsurvey-hb_<br>SOLEXA_5_FBgn0001180<br>M2; pos=3; reg=38<br>p.adj = 7.68 x 10^{-5}	flyfactorsurvey-Kr_<br>NAR_FBgn0001325<br>M2; pos=35; reg=27<br>p.adj= 0.0229	-	-
**KR**<br>**Paris *et al.* 2013**<br>(2149)<br>melanogaster group	transfac_pro-M01776<br>M2; pos=1; reg=70<br>p.adj = 3.96 x 10^{-11}	-	jaspar-MA0234.1<br>M2; pos=125; reg=32<br>p.adj= 0.012	-	-	flyfactorsurvey-Kr_<br>NAR_FBgn0001325<br>M2; pos=2; reg=45<br>p.adj = 3.29 x 10^{-10}	-	-
**S2 STARRseq**<br>**Arnold *et al.* 2014**<br>(1332)<br>*D.pseudoobscura*	elemento-ACATATG<br>M5; pos=1; reg=40<br>p.adj = 1.95 x 10^{-6}	-	-	-	-	-	-	-

Supplementary Figure 4: Divergent motif discovery comparing all models with the wAIC at once. All results for six datasets, including ChIP-seq binding in Drosophila embryo for Twist (He et al. 2011), BCD, GT, HB and KR (Paris et al. 2013) and functional enhancers specific for *D.pseudoobscura* detected by STARR-seq in S2 cells (Arnold et al. 2014). Green motifs indicate the expected motifs to be found on the diagonal (all correct except Giant). Red border marks all the Zelda CRMs gains and losses, only found for the embryonic ChIP peaks.

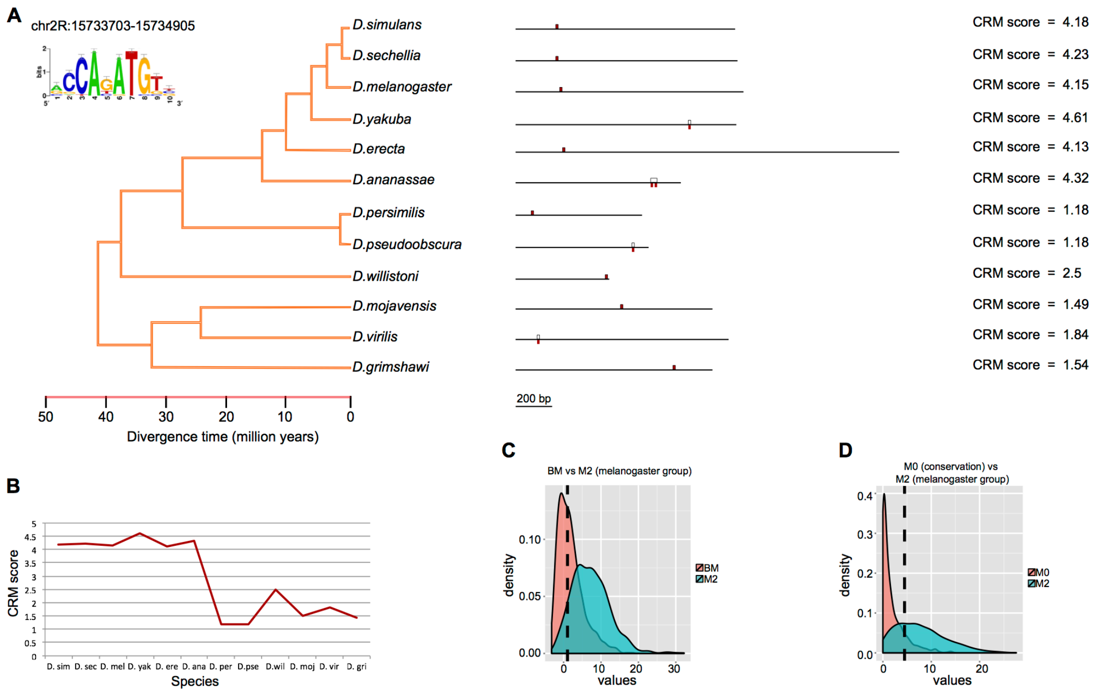

Supplementary Figure 5: Example of identifiability issues in model selection.

(A) Representation of the Drosophila phylogenetic tree with orthologous regions presenting Twist ChIP binding for the melanogaster group species, namely, *D.melanogaster*, *D.simulans*, *D.sechellia*, *D.yakuba*, *D.erecta* and *D.ananassae*. In red detected motifs corresponding to the CRM with maximum score in the region, and the respective CRM scores for twist PWM. (B) Graphical visualization of CRM scores for Twist across the Drosophila phylogeny. (C-E) Distribution of the likelihood ratio statistic for different model comparisons. The dashed vertical line indicates the observed value of likelihood ratio when the models are fit for the region of interest. (C-D) In each case the pink distribution shows the distribution of the likelihood ratio values obtained by bootstrapping under Brownian Motion (BM), while the blue distribution shows the distribution under the more complicated of the two models. (C) BM versus M0 (conservation); (D) BM versus M2 (melanogaster group). In this example, although figure B would clearly suggest a branch-specific shift, the different models cannot e unambiguously discriminated.

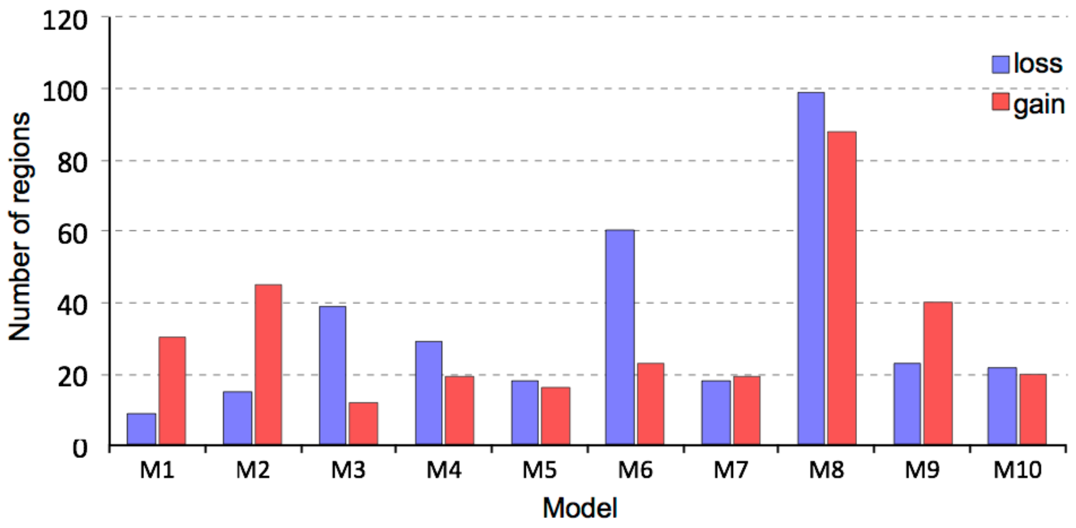

Supplementary Figure 6: Model choice; all branch-specific Twist ChIP-seq peaks in the melanogaster group, and their relative numbers of across the 9 lineage-specific evolutionary regimes using wAIC. We do not notice any strong bias of any branch-specific model, except perhaps a slightly higher number of wins for M8, the melanogaster-specific model. We do not entirely understand why there could be a slight bias towards this model, besides the better genome assembly and annotation that is available for this species. The “correct” evolutionary models, M1 and M2, contain a greater number of gains compared to losses for Twist CRMs, corresponding to the ChIP’d regions that are specific for the M1/M2 group.
